# An In Vitro Nutritional Evaluation of Mixed Silages of Drought‐Impaired Grass and Sugar Beet Pulp With or Without Silage Inoculants

**DOI:** 10.1111/jpn.14092

**Published:** 2025-01-06

**Authors:** Theresa Gruber, Claudia Lang, Katerina Fliegerová, Georg Terler, Qendrim Zebeli, Thomas Hartinger

**Affiliations:** ^1^ Centre for Animal Nutrition and Welfare University of Veterinary Medicine Vienna Austria; ^2^ Institute of Animal Physiology and Genetics Czech Academy of Sciences Prague Czech Republic; ^3^ Institute of Livestock Research Agricultural Research and Education Centre Raumberg‐Gumpenstein Irdning‐Donnersbachtal Austria

**Keywords:** anaerobic fungi, fibre degradation, rumen microbiota, ruminal fermentation, Rusitec, silage additive

## Abstract

Increasing droughts adversely affect grasslands, diminishing the availability and quality of forages for ruminants. We have recently shown that mixed ensiling of drought‐impaired grass (DIG) with sugar beet pulp (SBP) improved the conservation and feed value of silage. The application of silage additives may further improve the ruminal degradability, which may thereby shape the fermentation and microbiome in the rumen when those silages are tested as part of dairy diets. Therefore, we performed a long‐term in vitro nutritional evaluation of diets containing 50% (DM basis) of mixed silages from DIG and SBP, ensiled either with no additive (T_CON) or with anaerobic fungi culture supernatant (25% in DM; T_AF), mixed ruminal fluid (10% in DM; T_RF) or lactic acid bacteria (1% in FM; T_LAB). The data showed a high degradability of all diets (e.g., > 70% for organic matter), though without differences in nutrient degradabilities among treatments (*p* > 0.05). Fermentation characteristics, such as ruminal pH, short‐chain fatty acid profile, and gas production were only marginally affected by the treatments. Isobutyric acid proportion was higher in T_CON than in T_AF (*p* = 0.01), whereas isovaleric acid proportion was lower in T_LAB than in T_RF (*p* = 0.01). The analysis of the bacterial community revealed similar diversity and structure across all treatments in both the liquid and solid fraction. Noteworthy, *Lactobacillus* was among the predominant genera in the liquid fraction, which may have derived from the mixed silages. In conclusion, mixed silages from DIG and SBP as part of a 50% concentrate diet showed high ruminal degradability, but no beneficial impact by the tested silage additives was observed. Hence, under these conditions, their application appears not justified. Our results warrant further in vivo verification, whereby it would be of interest to determine the impact of the applied silage additives in forage‐based diets (e.g., > 50% silage in diet DM) in future research.

## 1 Introduction

Global climate patterns are changing and extreme weather phenomena have become more frequent in recent decades, including prolonged droughts, high temperatures or massive rain falls (Forzieri et al. 2014). Such weather events, especially drought events, have a significant impact on the grassland (Ciríaco da Silva et al. 2013). Under such drought conditions, plants cannot grow sufficiently, which leads to feed shortages, diminished nutritive quality and low palatability of forages (Rare 1990). Indeed, in our latest research, we observed that drought‐impaired grass (DIG) was characterized by low crude protein (CP) levels (99 g/kg DM) and high fibre concentrations, particularly cellulose and lignin (249 and 76 g/kg DM, respectively), which led to low energy concentrations (Gruber et al. 2024).

Despite these challenges, ruminants need adequate amounts and qualities of forages, with grass silages being one of the main sources of energy, protein and physically effective fibre in the diet. A sufficient quality of grass silage is especially important since deficiencies in feed value cannot be arbitrarily compensated with starch‐rich feeds due to the risk of (sub)acute rumen acidosis and its detrimental consequences for animal health (Zebeli et al. 2012). To overcome these limitations of DIG, mixed ensiling with local by‐products, such as sugar beet pulp (SBP) was shown to successfully enhance the feed value and conservation properties in the silages, while simultaneously providing physically effective fibre to cattle (Gruber et al. 2024).

While mixed ensiling of DIG substantially enhances the nutritive value, novel silage additives, including anaerobic fungi culture supernatant (AF; Hartinger, Fliegerová, and Zebeli 2022) may further improve silage degradability and alter the fermentation profile in the rumen. For instance, the addition of AF during ensiling of whole‐crop corn and grass silages led to more lactic acid, hence contributing to a better energy conservation (Hartinger et al. 2024; Gruber et al. 2024). The AF inoculation also resulted in a higher fibre degradability for whole‐crop corn silages (Hartinger et al. 2024), whereas these beneficial effects were not continuously present in grass silages (Hartinger, Fliegerová, and Zebeli 2022; Gruber et al. 2024). However, all those observations were made in short‐term batch culture incubations, which suffer from the accumulation of metabolites and a steep decline in microbial viability already after few hours. This fact can be a particular limitation when studying dietary interventions in the rumen, because alterations can develop over days or even weeks rather than hours (Czerkawski and Breckenridge 1977). In this context, the rumen simulation technique (Rusitec) offers an in vitro long‐term examination of the dietary effects.

Apart from AF, the suitability of mixed ruminal fluid (RF) as a silage additive was recently examined as it harbours a vast number of fibre‐cleaving enzymes and metabolically active microorganisms that together can effectively degrade plant cell wall structures (Li et al. 2020). The application of RF during ensiling caused slightly higher in vitro gas productions in mixed silages (Gruber et al. 2024; Hartinger et al. 2024). Thus, the closer examination during long‐term in vitro rumen fermentation appears prudent. The impact of conventional microbial inoculants like lactic acid bacteria (LAB) on the rumen fermentation is poorly understood and contradictory research outcomes (Ellis et al. 2016) demand for further investigations.

The aim of the study was to analyze the long‐term in vitro rumen fermentation and nutrient degradabilities of diets with mixed silages of DIG and SBP, prepared either with no additive or with AF, RF or LAB. Based on the fibre‐cleaving impact by fungal enzymes during ensiling (Hartinger and Zebeli 2021), the hypothesis was that diets including AF and RF‐treated silages have a higher in vitro fibre degradability than control diets. Since there are indications that the microorganisms present in silages can modulate the ruminal microbiome (e.g., Hartinger et al. 2019), we further expected that the silage inoculants differently shape the bacterial community during the long‐term in vitro incubation.

## 2 Materials and Methods

### 2.1 Experimental Design and Treatment Diets

The present experiment was conducted in a completely randomized design with four different treatment diets. The treatment diets were incubated in a Rusitec system over two independent runs with three fermenters per treatment per run, thus resulting in *n* = 6 per treatment.

Each treatment diet consisted of 50% concentrate mixture and 50% mixed silage (DM basis) with the mixed silages being different between the treatment diets. The exact preparation of the mixed silages was described in detail in Gruber et al. (2024) and their chemical composition plus silage fermentation characteristics are stated in Supporting Information S1: Table 1. Briefly, the silages were prepared from DIG and SBP pellets and mixed on a DM basis in a ratio of 63% of DIG and 37% of SBP. Directly before ensiling, the mixtures were treated with either (i) no additive as control, (ii) fresh AF (25% in DM), (iii) fresh RF (10% in DM) or (iv) LAB (1% in FM, corresponding to 250,000 colony‐forming units/g FM). Detailed information on the preparation of the silage additives can be obtained from our companion publication (Gruber et al. 2024). For the present experiment, the silages were chopped manually to a particle length of 3–4 cm.

The concentrate mixture comprised 34% of commercially available protein concentrate pellets (Rindastar 39 XP; H. Wilhelm Schaumann GmbH & Co KG, Brunn am Gebirge, Austria) and 66% of ground grain mixture (Table 1). It was ground to pass a 6 mm sieve (ZM 200, Retsch, Haan, Germany) and their chemical compositions are illustrated in Supporting Information S1: Table 2. Based on the different silage additives applied in the mixed silages, the four treatment diets were denoted as [T_CON], [T_AF], [T_RF] and [T_LAB], respectively, and their exact ingredients and chemical compositions are given in Table 1.

**Table 1:** Ingredients and chemical composition of treatment diets.

	Treatment diet^a^			
	T_CON	T_AF	T_RF	T_LAB
Constituents of treatment diets (% DM^b^)				
Mixed silage without additive	50	0	0	0
Mixed silage with anaerobic fungi culture supernatant	0	50	0	0
Mixed silage with mixed ruminal fluid	0	0	50	0
Mixed silage with lactic acid bacteria	0	0	0	50
Ground grain mixture^c^	33	33	33	33
Protein concentrate pellets^d^	17	17	17	17
Chemical composition (g/kg DM, unless otherwise noted)				
DM concentration (g/kg)	627	626	630	624
Ash	85.5	86.8	85.5	85.6
Crude protein	167	167	165	164
Ether extract	22.9	23.2	21.6	22.4
aNDFom^e^	360	344	353	343
ADFom^f^	214	230	213	218
ADL^g^	50.5	80.7	48.2	49.0
Nonfiber carbohydrates	364	378	375	385

^a^ Diets consisting of 50% concentrate and 50% of mixed silage from drought‐impaired grass and sugar beet pulp pellets without an additive (T_CON), with fresh anaerobic fungi culture supernatant (T_AF), with fresh mixed ruminal fluid (T_RF) or with lactic acid bacteria (T_LAB).

^b^ Dry matter.

^c^ Composition on DM basis: 50% ground corn, 21% ground barley, 21% ground wheat, 3% limestone, 5% Rindavit TMR 11 ASS‐Co+ATG (Schaumann GmbH & Co KG, Germany).

^d^ Rindastar 39 XP; H. Wilhelm Schaumann GmbH & Co KG, Brunn am Gebirge, Austria.

^e^ Neutral detergent fibre assayed with a heat‐stable α‐amylase and expressed exclusive of residual ash.

^f^ Acid detergent fibre expressed exclusive of residual ash.

^g^ Acid detergent lignin.

### 2.2 Rusitec Procedure

Each Rusitec run lasted 10 days, with the first 5 days used for adaptation and the last 5 days for sampling. In each run, 12 fermenters were used, and every fermenter had a volume of about 700 mL. The implementation and daily procedure of the Rusitec system was in accordance with the protocol of Khiaosa‐ard et al. (2015). In brief, the experimental buffer (McDougall 1948) was constantly infused at a rate of 326 ± 19.2 mL/d using a 12‐channel peristaltic pump (Model ISM 932D; Ismatec, IndexHealth and Science GmbH, Wertheim, Germany). The effluent bottles were kept at 1°C in a refrigerator and all fermentation gases were collected in gas‐tight bags (TECOBAG 8 L; Tesseraux Container GmbH, Bürstadt, Germany). On the first day of each run, ruminal content was collected from two nonlactating, rumen‐fistulated Holstein cows of the Clinical Centre for Ruminant and Camelid Medicine, University of Veterinary Medicine Vienna, which were fed grass hay ad libitum and additionally received one kg of concentrate (KuhKorn PLUS Energie; Garant‐Tiernahrung GmbH, Pölchlarn, Austria) per cow per day. The RF was pooled and subsequently filtered through four layers of gauze (Wilhelm Weisweiler GmbH & Co. KG, Münster, Germany), the solid ruminal content was pooled and about 60 g was filled in a fresh nylon bag (100 × 200 mm, 50 µm pore size; R1020, ANKOM Technology, Macedon, NY, USA). Two nylon bags, one with solid rumen content and one with the respective treatment diet were placed into the fermenters. The nylon bags with the treatment diets contained 12 g DM of the respective diet, freshly prepared right before the daily feedbag exchange. Hereby, all diet components, that is, the mixed silage and both concentrate sources, were weighed separately into the nylon bags. Then, every fermenter was filled with 600 mL RF and 100 mL of buffer solution. On the next day, the nylon bags containing the solid rumen digesta were replaced by nylon bags containing the respective treatment diets. On the following days, the nylon bags that had remained in the fermenter for 48 h were replaced by new nylon bags containing the fresh diets, resulting in a 48 h incubation period for each nylon bag.

### 2.3 Sample Collection

During the sampling period, fermenter fluid was sampled daily from each fermenter before exchanging the nylon bags. The pH and redox potential were directly measured using a pH meter equipped with two separate electrodes for pH and redox potential (S40‐K SevenMult, Mettler Toledo, Vienna, Austria). Likewise, aliquots for short‐chain fatty acids (SCFA) and ammonia‐N analysis were taken from the fermenter fluid and kept at −20°C. The aliquots for the bacterial community analysis were immediately snap frozen in liquid nitrogen and afterward stored at −20°C. The total gas volume of each fermenter was determined daily using the water replacement technique (Soliva and Hess 2007) and a portable infrared detector (ATEX Biogas Monitor Check BM 2000, Ansyco, Karlsruhe, Germany) was used for measuring the gas composition. The nylon bags removed from the fermenters were washed with cold water and no soap for 30 min in a washing machine as described by Khiaosa‐ard et al. (2023), and then stored at −20°C. The content of the nylon bags, which were obtained on the last day of each run and, therefore, were only incubated for 24 h were directly snap frozen in liquid nitrogen and then stored at −20°C for subsequent utilization in bacterial community analysis.

### 2.4 Sample Analyses

The diet residues were freeze‐dried for 24 h and afterward ground using a 0.5 mm sieve in an ultra‐centrifugal mill (ZM 200, Retsch, Haan, Germany). The analysis of chemical composition of diet residues were carried out following the instructions of the Association of German Agricultural Analytic and Research Institutes (VDLUFA 2012). For determining the DM concentration, the samples were dried at 103°C for a minimum of 4 h (method 3.1). The ash concentration was determined by combustion in a muffle furnace at 580°C for at least 4 h (method 8.1). With the Kjeldahl method (method 4.1.1) CP was analyzed, and ether extract (EE) was determined using the Soxhlet extraction system (Extraction System B‐811, Büchi, Flawil, Switzerland; method 5.1.2). Determination of neutral detergent fibre assayed with a heat‐stable α‐amylase and expressed exclusive of residual ash (aNDFom), acid detergent fibre expressed exclusive of residual ash (ADFom) and acid detergent lignin (ADL) were carried out by methods 6.5.1, 6.5.2 and 6.5.3, respectively. The WSC concentration was analyzed following the method 7.1.1., the concentration of nonfiber carbohydrates (NFC) was calculated using the formula NFC = 1000 − (CP + ash + EE + NDF), all values in g/kg DM. The 48 h degradability of each nutrient was calculated by dividing the concentration of degraded nutrients (difference between the nutrient amount present in the diet before incubation and the amount recovered in the diet residue after 48 h of incubation in the Rusitec system) by the nutrient concentration in the diet before incubation. The analyses of SCFA and ammonia‐N concentrations were carried out by gas chromatography and based on the Berthelot reaction (Hinds and Lowe 1980), respectively, as described before in Gruber et al. (2024).

### 2.5 Analysis of Bacterial Community Composition

#### 2.5.1 DNA Extraction and Sequencing

Sequencing of the 16S rRNA gene was performed on the liquid and solid fraction. Therefore, DNA was extracted from about 800 µL of fermenter fluid and 250 mg of solid content using the DNeasy PowerSoil Pro Kit (Qiagen, Hilden, Germany) in accordance with the manufacturer's protocol and with the addition of mutanolysin, lysozyme and proteinase K. Additionally, DNA extracts of blank controls of each run were included in analysis to prevent potential bias from cross‐contamination during the DNA extraction procedure (Hornung, Zwittink, and Kuijper 2019). After the isolation, DNA quantity was determined using a fluorometer (Qubit Fluorometer 2.0, Thermo Fisher Scientific, Austria) and the respective kit (Qubit dsDNA HS Assay Kit, Thermo Fisher Scientific, Austria) by following the manufacturer's instructions. The hypervariable region V4 of the 16S rRNA gene (2 × 250 bp) was amplified using the primer pair 515 F (5′‐GTGCCAGCMGCCGCGGTAA‐3′) and 806 R (5′‐GGACTACHVGGGTWTCTAAT‐3′). The gene sequencing was performed on the NovaSeq. 6000 sequencing platform and demultiplexing and trimming of adapters and primers was also done by Novogene (Novogene Co. Ltd, Cambridge, United Kingdom).

#### 2.5.2 Bioinformatic Analysis

The sequencing data set was processed using the software Quantitative Insights into Microbial Ecology QIIME2 v2024.5 (Bolyen et al. 2019). The read quality was inspected using FASTQC with the PHRED score offset of 33. Stitching of reads, quality filtering and denoising into amplicon sequence variants (ASV) was done using DADA2 (Callahan et al. 2016). Moreover, decontam (Davis et al. 2018) was used to identify contaminants considering the data of blank controls (prevalence) and the DNA concentrations of both samples and blank controls (frequency). Then, these contaminants were excluded from representative sequences and feature tables using the default threshold of 0.1, together with mitochondria and chloroplast. The resulting ASV were aligned with mafft (Katoh et al. 2002) and a phylogeny was constructed with FastTree2 (Price, Dehal, and Arkin 2010). Taxonomy was assigned to ASV using a classify‐sklearn naïve Bayes taxonomy classifier trained with the 515 F/806 R primer set against the SILVA Small Subunit rRNA database v138 (Quast et al. 2012). Afterward, the filtered feature table, rooted tree and taxonomy were imported in RStudio v2024.04.1 for further analysis.

### 2.6 Statistical Analyses

Except for bacterial community composition data, all statistical analyses were performed in SAS (version 9.4; SAS Institute Inc., Cary, NC, USA). First, the data was analyzed for outliers using PROC REG and values were excluded if the adjusted Cook's D threshold was not met. Then, the Shapiro–Wilk's normality method of PROC UNIVARIATE was used to validate normal distribution. When the data of a specific variable were not normally distributed, they were logarithmically transformed or, if needed, square root transformed in a second step. Afterward, all data were analyzed in a variance analysis according to the completely randomized group design using PROC MIXED, including the fixed effect of the treatment diet and experimental run as random effect. The first‐order autoregressive structure with heterogeneous variances was used to account for repeated measurements in the same experimental unit within a fermenter. The degrees of freedom were approximated according to Kenward and Roger (1997). The post hoc multiple mean comparisons were carried out using the Tukey–Kramer test. The significance level was defined at *p* ≤ 0.05 and trends were declared at 0.05 < *p* < 0.10.

**Figure 1:**
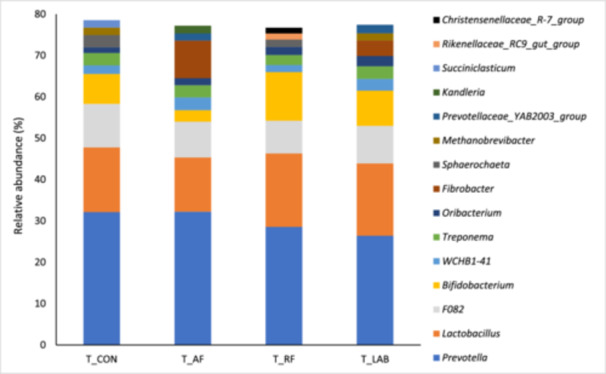
Relative abundances of the 10 most abundant genera in the liquid fraction when incubating diets differing in silages prepared from drought‐impaired grass and sugar beet pulp pellets without an additive (T_CON), with fresh anaerobic fungi culture supernatant (T_AF), with fresh mixed ruminal fluid (T_RF) or with lactic acid bacteria (T_LAB). [Color figure can be viewed at wileyonlinelibrary.com]

**Figure 2:**
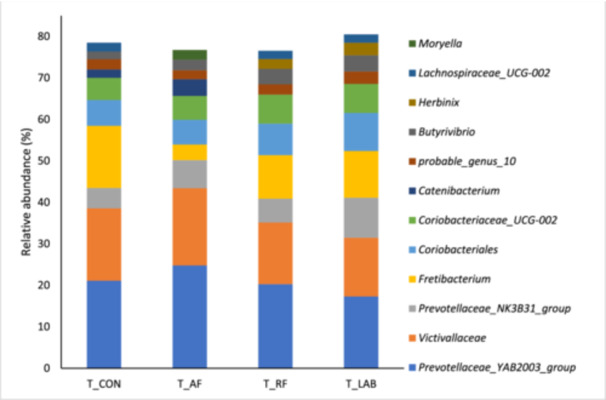
Relative abundances of the 10 most abundant genera in the solid fraction when incubating diets differing in silages prepared from drought‐impaired grass and sugar beet pulp pellets without an additive (T_CON), with fresh anaerobic fungi culture supernatant (T_AF), with fresh mixed ruminal fluid (T_RF) or with lactic acid bacteria (T_LAB). [Color figure can be viewed at wileyonlinelibrary.com]

Regarding the bacterial community composition, data of the liquid and solid fraction were analyzed separately, as the fractions showed a distinctly different structure (Supporting Information S1: Figures 1 and 2), which is in accordance with observed Rusitec studies (e.g., Hartinger et al. 2019). The alpha diversity metrics were calculated in RStudio and then imported in SAS for statistical analysis using the PROC MIXED model as described above. Except for alpha diversity metrics, sequencing data were analyzed in RStudio. The differences in beta diversity were calculated using the vegan package and the *adonis2* function (Anderson 2001) and principal coordinates analysis (PCoA) plots were created using weighted UniFrac distance, unweighted UniFrac distance and Bray‐Curtis distance metrics. The differential abundances at genus level were calculated using the package MaAsLin2 (Mallick et al. 2021). Thereby, changes in abundances were considered as relevant if coefficient was < −2.00 or > 2.00 and Benjamini‐Hochberg false discovery rate‐adjusted q‐values were < 0.05. The Venn diagrams were created with the tool Venny v2.1 (Oliveros 2007–2015) to determine bacterial genera that were exclusively present in specific treatment diets.

## 3 Results

### 3.1 In Vitro Fermentation Characteristics

Table 2 presents data of the ruminal fermentation characteristics, showing that the fermenter pH varied from 6.23 to 6.26 and also total SCFA ranged from 129 to 134 mmol/l with acetate (49.2% ± 0.3), propionate (27.6% ± 0.3) and then n‐butyrate (13.0% ± 0.5) being the dominant SCFA. No differences were observed among treatments, except for the proportions of both branched‐chain fatty acids. For isobutyric acid, the highest percentage was found in fermenters incubated with T_CON, which was higher than for T_AF that had lowest isobutyric acid proportions for T_AF (*p* = 0.01). Likewise, the percentage of isovaleric acid ranged between 3.99% and 4.30% of total SCFA and was lowest during incubations of T_LAB and highest with T_RF (*p* = 0.01). Furthermore, the ammonia‐N concentration tended to be higher in T_CON than in T_AF with T_RF and T_LAB in the middle (*p* = 0.07). Apart from these variables, no impact of treatment diets was found for other variables related to in vitro ruminal fermentation (*p* > 0.10), such as pH, redox potential or total SCFA concentration. Likewise, the daily production of total fermentation gases, as well as methane and carbon dioxide were not different between treatment diets (*p* > 0.10). A tendency was found when relating the daily methane production to the amount of degraded aNDFom (*p* = 0.08), but the post hoc test revealed no differences between treatment diets. Relating the daily methane production to the amount of degraded OM showed no impact by the treatments (*p* > 0.10).

**Table 2:** Effect of treatment diets on in vitro ruminal fermentation characteristics and gas composition.

	Treatment^a^					
	T_CON	T_AF	T_RF	T_LAB	SEM^b^	*p* value
pH	6.25	6.24	6.26	6.23	0.04	0.95
Redox (mV)	−262	−258	−269	−265	−8.17	0.82
Total gas (mL/d)	4078	4252	3866	4121	141	0.17
Methane (mL/d)	159	165	144	159	9.09	0.16
Methane (mL/g degraded OM^c^)	18.3	19.0	17.1	18.7	1.01	0.25
Methane (mL/g degraded aNDFom^d^)	30.1	32.1	28.7	32.5	2.51	0.08
Carbon dioxide (mL/d)	1132	1184	1050	1136	50.4	0.26
Ammonia‐N (mmol/l)	8.75	7.97	8.64	8.62	0.24	0.07
Total SCFA^e^ (mmol/l)	134	129	130	133	12.1	0.86
Acetic acid (%)	49.1	49.3	48.8	49.6	0.87	0.70
Propionic acid (%)	27.8	27.1	27.5	27.8	1.95	0.38
Butyric acid (%)	13.2	13.3	13.2	12.2	0.98	0.27
Isobutyric acid (%)	0.64^A^	0.46^B^	0.59^A,B^	0.55^A,B^	0.05	0.01
Valeric acid (%)	3.60	3.79	3.62	3.79	0.20	0.69
Isovaleric acid (%)	4.22^A,B^	4.16^A,B^	4.30^A^	3.99^B^	0.23	0.01
Caproic acid (%)	1.62	1.80	1.56	1.63	0.09	0.10
Heptonic acid (%)	0.23	0.21	0.22	0.24	0.00	0.87
Acetate/propionate	1.80	1.85	1.79	1.81	0.16	0.68
Acetate + butyrate/propionate + valerate	2.00	2.06	2.00	1.97	0.18	0.24

*Note:* In each row, different capitalized superscript letters indicate significant difference between least square means (*p* ≤ 0.05).

^a^ Diets consisting of 50% concentrate and 50% of silage prepared from drought‐impaired grass and sugar beet pulp pellets without an additive (T_CON), with fresh anaerobic fungi culture supernatant (T_AF), with fresh mixed ruminal fluid (T_RF) or with lactic acid bacteria (T_LAB).

^b^ Standard error of the mean.

^c^ Organic matter.

^d^ Neutral detergent fibre assayed with a heat‐stable α‐amylase and expressed exclusive of residual ash.

^e^ Short‐chain fatty acids.

### 3.2 Nutrient Degradability

Table 3 shows the data of 48 h degradability of nutrients, showing that the degradability of DM varied from 71.2% to 72.2% and OM degradability ranged from 71.0% to 71.8%. The degradability of CP and aNDFom ranged from 76.2% to 81.4% and from 40.8% to 47.4%, respectively. The highest degradabilities were observed for NFC and lowest for ADFom, ranging from 94.2% to 96.4% and 37.3% to 40.3%, respectively. The ANOVA indicated that the degradability of nutrients was not different among the treatment diets (*p* > 0.05). This was true for all proximate nutrients as well as for aNDFom and ADFom, plus the calculated fractions OM and NFC.

**Table 3:** Effect of treatment diets on the 48 h in vitro nutrient degradabilities (%).

	Treatment^a^					
	T_CON	T_AF	T_RF	T_LAB	SEM^b^	*p* value
Dry matter	72.2	71.5	71.6	71.2	1.62	0.98
Organic matter	71.7	71.0	71.1	71.8	1.69	0.98
Ash	77.1	77.0	76.1	76.7	0.56	0.29
Crude protein	81.4	78.6	77.8	76.2	2.17	0.41
Ether extract	76.7	74.5	75.6	73.0	3.46	0.83
aNDFom^c^	47.4	41.3	42.9	40.8	3.41	0.29
ADFom^d^	38.8	40.3	37.4	37.5	2.93	0.85
Nonfiber carbohydrates	96.3	95.9	94.7	94.1	1.21	0.49

^a^ Diets consisting of 50% concentrate and 50% of silage prepared from drought‐impaired grass and sugar beet pulp pellets without an additive (T_CON), with fresh anaerobic fungi culture supernatant (T_AF), with fresh mixed ruminal fluid (T_RF) or with lactic acid bacteria (T_LAB).

^b^ Standard error of the mean.

^c^ Neutral detergent fibre assayed with a heat‐stable α‐amylase and expressed exclusive of residual ash.

^d^ Acid detergent fibre expressed exclusive of residual ash.

### 3.3 Bacterial Community Composition

The final data set comprised 7,668,171 reads. In the liquid fraction, seven of the 10 most abundant genera were present in all treatments with *Prevotella* and *Lactobacillus* accounting for around 45% of all present genera in all treatments (Figure 1). The ‘top ten’ genera were the same in all treatments except for four genera: *Kandleria* was only present with T_AF, *Succiniclasticum* with T_LAB, and *Rikenellaceae* RC9 gut group and Christensenellaceae R‐7 group were only among the 10 most abundant genera during T_RF incubation. Moreover, the 10 predominant genera represented the vast majority of all reads in all treatments, i.e., about 77% (Figure 1). In the solid fraction, *Prevotellaceae*_YAB2003_group and *Victivallaceae* were the most abundant genera in all treatment diets, amounting for 31% to 43% of the reads (Figure 2). The ‘top ten’ genera were the same in all treatments except for four genera, which were *Catenibacterium* (in T_AF and T_CON), *Herbinix* (in T_LAB and T_RF), *Lachnospiraceae_UCG‐002* (in T_CON, T_LAB and T_RF) and *Moryella* (in T_AF). Nevertheless, the 10 predominant genera stand for the majority of all reads in all treatments, that is, about 78%, also in the solid fraction (Figure 2).

**Figure 3:**
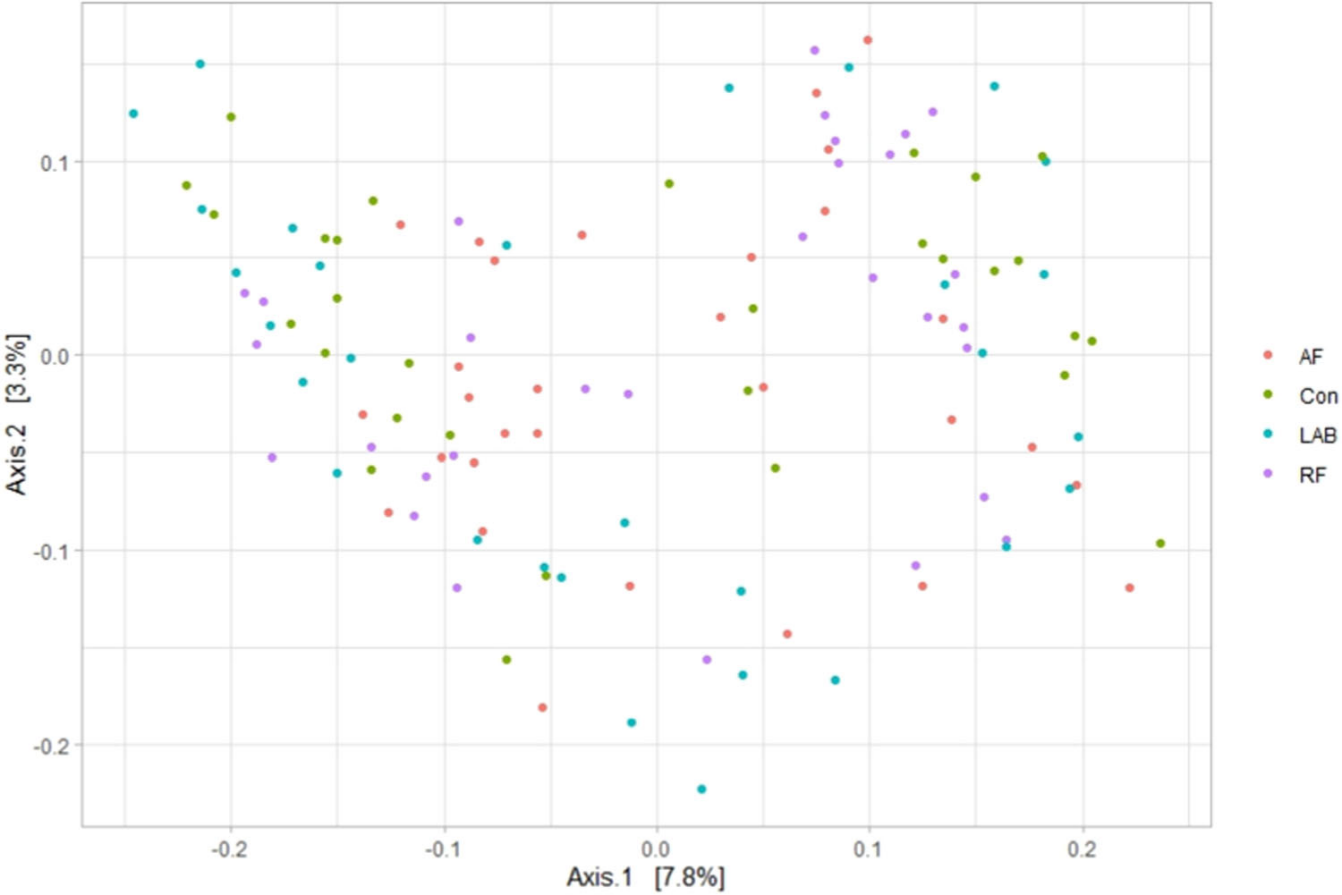
Changes in bacterial community composition in the liquid fraction associated with different treatment diets visualized as a principal co‐ordinate analysis using weighted UniFrac distance metrics. The percentage of variation is indicated on the respective axes. Treatment diets differed in the included silage of drought‐impaired grass and sugar beet pulp pellets produced (i) without additive [CON], (ii) with fresh anaerobic fungi culture supernatant [AF], (iii) with fresh mixed ruminal fluid [RF] or (iv) with lactic acid bacteria [LAB]. [Color figure can be viewed at wileyonlinelibrary.com]

**Figure 4:**
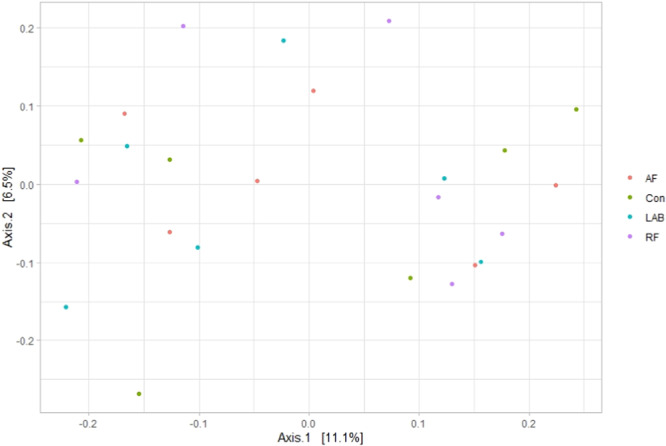
Changes in bacterial community composition in the solid fraction associated with different treatment diets visualized as a principal co‐ordinate analysis using weighted UniFrac distance metrics. The percentage of variation is indicated on the respective axes. Treatment diets differed in the included silage of drought‐impaired grass and sugar beet pulp pellets produced (i) without additive [CON], (ii) with fresh anaerobic fungi culture supernatant [AF], (iii) with fresh mixed ruminal fluid [RF] or (iv) with lactic acid bacteria [LAB]. [Color figure can be viewed at wileyonlinelibrary.com]

**Figure 5:**
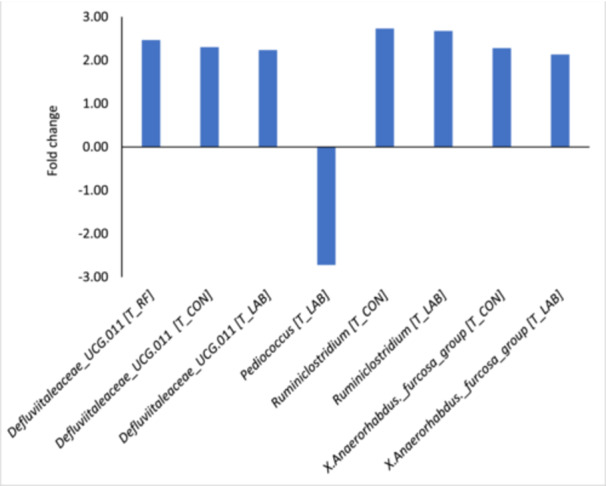
Differential abundance of bacterial genera in the liquid fraction when incubating diets differing in silages prepared from drought‐impaired grass and sugar beet pulp pellets without an additive (T_CON), with fresh anaerobic fungi culture supernatant (T_AF), with fresh mixed ruminal fluid (T_RF) or with lactic acid bacteria (T_LAB). The treatment with anaerobic fungi culture supernatant was used as benchmark. [Color figure can be viewed at wileyonlinelibrary.com]

As illustrated in Table 4, the alpha diversity metrics were not different between the treatment diets (*p* > 0.05). Thus, neither the richness nor the diversity of the bacterial community were affected. Based on the weighted UniFrac distance, the beta diversity showed no clustering of the data according to the treatment effect, which was true for both the liquid (*p *= 0.06; Figure 3) and the solid fraction (*p *= 0.80; Figure 4). It is noteworthy that despite the tendency for an influence of treatment diets in the liquid fraction, no clear pattern was found (Figure 3). Similarly, the variations explained on the horizontal and vertical PCoA axes were generally low, that is, 7.8% and 3.3% in liquid fraction and 11.1% and 6.5% in the solid fraction (Figures 3 and 4). In addition, the analysis using unweighted UniFrac or Bray–Curtis distance metrics also revealed no differences in beta diversity structure for the liquid and solid fraction (Supporting Information S1: Figures 3–6).

**Table 4:** Effect of treatment diets on alpha diversity metrics in the liquid and solid fraction.

	Treatment^a^					
	T_CON	T_AF	T_RF	T_LAB	SEM^b^	*p* value
Liquid fraction						
Observed ASV^c^	1193	1112	1137	1161	9.27	0.52
Shannon	4.27	4.17	4.19	4.33	0.02	0.59
InvSimpson	18.5	18.9	19.1	17.9	0.46	0.97
Fisher	219	204	210	216	2.00	0.60
Solid fraction						
Observed ASV	690	679	760	677	31.7	0.68
Shannon	3.83	3.77	3.83	3.92	0.10	0.94
InvSimpson	19.9	15.6	19.5	20.1	1.49	0.71
Fisher	117	115	130	114	6.31	0.70

^a^ Diets consisting of 50% concentrate and 50% of silage prepared from drought‐impaired grass and sugar beet pulp pellets without an additive (T_CON), with fresh anaerobic fungi culture supernatant (T_AF), with fresh mixed ruminal fluid (T_RF) or with lactic acid bacteria (T_LAB).

^b^ Standard error of the mean.

^c^ Amplicon sequence variants.

The determination of differentially abundant genera identified four genera in the liquid fraction, which are illustrated in Figure 5, whereas no differently abundant genera were found in the solid fraction. The genus *Pediococcus* was lower abundant in T_LAB compared to T_AF (coefficient −2.72), the genus *Defluviitaleaceae* UCG.011 was higher abundant in T_CON (coefficient 2.30), T_RF (coefficient 2.47) and T_LAB (coefficient 2.23) compared to T_AF. Moreover, *Ruminoclostridium* and *X. Anaerorhabdus* furcosa group were higher abundant in T_CON (coefficients 2.73 and 2.28, respectively) and T_LAB (coefficients 2.68 and 2.13, respectively) than in T_AF.

Similarly, the Venn diagrams revealed that the vast majority of genera were shared between all treatments in the liquid fraction, that is, 218 genera or 76.5% (Supporting Information S1: Figure 7). Only 5.3%, 2.8%, 2.5% and 4.6% were exclusively present in T_CON, T_AF, T_RF and T_LAB treatments, respectively (Supporting Information S1: Figure 7). A similar pattern was observed in the solid fraction, where 65.9% of all bacterial genera (147 genera) were present in all treatments and only 4.5% were exclusively in T_CON, 2.7% only in T_AF, 7.2% in T_RF and 4.5% T_LAB (Supporting Information S1: Figure 8).

## 4 Discussion

The lacking impact of silage additives on nutrient degradability was unexpected. The application of RF and in particular AF during ensiling of grass and whole‐crop corn resulted in an improved in situ or short‐term in vitro fibre degradability, when these silages were incubated alone (Hartinger, Fliegerová, and Zebeli 2022; Gruber et al. 2024; Hartinger et al. 2024). Therefore, an increased degradability of aNDFom and ADFom was hypothesized when incubating mixed silages produced with AF or RF inoculants as part of the treatment diet with 50% concentrate in the Rusitec system. However, based on our data, this hypothesis was not confirmed.

One explanation could be different fibre (NDF) degradation rates of forages in the rumen. Weisbjerg, Koukolová, and Lund (2007) found a ruminal degradation rate of grass of up to 0.07/h in the first 20 h of incubation, whereas the degradation rate of corn and cereal whole crops did not exceed 0.03/h (Weisbjerg, Koukolová, and Lund 2007). Therefore, it may be that using AF as a silage additive in grass is less effective for enhancing fibre degradability than in corn or whole crop cereals. The lacking impact of the AF treatment in the present study may hence rather be related to a certain degree of substrate specificity. Nevertheless, the numerical increase of ADFom degradability by 2–3 percentage points in T_AF compared to the other treatments may still suggest a positive impact on ruminal fibre degradability. This is also supported by the numerically highest daily total gas and methane production for T_AF. As the degradability of the ADFom but not aNDFom fraction was higher for T_AF, it suggested that cellulose and lignin were more degradable in the AF‐treated mixed silages but not hemicelluloses. This observation on fibre degradability matches prior degradability data from in situ incubations of AF‐treated grass silages (Hartinger, Fliegerová, and Zebeli 2022) and indeed, AF were recently proven to deconstruct grass lignin anaerobically (Lankiewicz et al. 2023). It has to be mentioned that the present diets included 50% concentrate. Together with the SBP from the mixed silages, this means an indeed high provision of easily fermentable carbohydrates to the rumen microbes. This may have masked the effect of the silage additives on nutrient degradability and rumen fermentation pattern. Hence, it may be prudent to increase the diet's silage proportion in future research.

The SCFA profile, gas production and other fermentation characteristics were not different among treatments. This result corroborates with our findings of nutrient degradability. The high propionate proportions of on average 27.6 ± 0.3% could partly originate from fermentation but in parts also from metabolizing silage‐derived lactic acid that amounted for 11%–13% in all mixed silages (Gruber et al. 2024). Only the branched‐chain fatty acid proportions were different between certain treatments. Thereby, isobutyric acid was statistically and isovaleric acid was numerically lower in T_AF than in T_CON. As both branched‐chain fatty acids are formed during ruminal deamination (Carro and Miller 1999), this observation could be related to the tendency of lower ammonia‐N concentrations found in T_AF compared to T_CON.

Additionally, the lacking impact of the LAB treatment may also be explained by the fact that LAB inoculants are primarily designed to improve the preservation success in silages (Muck et al. 2018). The effectiveness of LAB‐based additives to influence the rumen fermentation, however, strongly depends on strain, dose as well as forage type (Ellis et al. 2016), which may have been not suitable for this purpose in the present study. Indeed, only the isovaleric acid proportion was 0.3% points lower in T_LAB than in T_RF, which appears negligible and no effects on any other variable were present.

Regarding the bacterial community composition, no significant differences were observed between the treatments in the liquid and the solid fraction. At the feedstuff level, inoculating silages with AF led to a clear shift in the silages' bacterial community composition, characterized by a severely lower diversity than observed with no additive, RF or LAB (Gruber et al. 2024). It appears that this lowering effect of the AF inoculant was not transferred from the silages to the rumen, so that our second hypothesis that the silage additives may differently shape the bacterial community composition was also not confirmed. Only the circumstance that, except for one genus, all differently abundant genera in the liquid fraction were found to be less present with T_AF than with other treatments may give a slight hint of the reducing force of the AF additive.

Presumably, the stability and resilience of the rumen microbiome prevailed over the present treatments (Weimer 2015), particularly as the different silage additives represented rather small nuances when compared to strong interventions like antibiotics. Still, the abundance of a few genera changed with the treatments, such as *Moryella* being predominant in T_AF but not in other treatments. Yet, there is only sparse information available for this genus, which is assumed to be a core member of the rumen microbiome (Romanzin et al. 2024). Considering the very similar in vitro fermentation pattern between all treatments, the differential abundance of *Moryella* may be of limited biological relevance.

It was surprising that *Lactobacillus* was the second most abundant genera for all treatments in the liquid fraction. Unless cattle are not suffering from rumen acidosis, *Lactobacillus* commonly constitutes a low abundant genus in the rumen (Nocek 1997; Henderson et al. 2015), which is also true for in vitro simulations like the Rusitec system (Khiaosa‐ard et al. 2015; Humer et al. 2018). However, lactobacilli can become dominant in Rusitec fermenters when high silage amounts are incubated as seen with alfalfa silages (Hartinger et al. 2019). In the mixed silages of our study, *Lactobacillus* constituted by far the predominant genus with a relative abundance of > 67% (Gruber et al. 2024). A transfer from the diet and the successful establishment of this genus in the Rusitec system—at least as long it was continuously reintroduced with the diet—appears, therefore, a plausible explanation. In addition, the high concentrate proportion in the diets should have contributed to the proliferation of *Lactobacillus* by providing sufficient metabolizable substrate (Yang et al. 2018; Zhang et al. 2020; Catunda et al. 2022).

## 5 Conclusion

The present long‐term in vitro study showed marginal differences between treatment diets and no additional effects by the silage additives used during production of mixed silages. However, overall high nutrient degradabilities and fermentative activity when incubating diets containing 50% mixed silages and 50% concentrate were observed. Similarly, the bacterial community composition in the liquid and solid fraction was only marginally affected by the treatment diets. Therefore, under these conditions, we found no benefits that would advocate the application of those silage additives in mixed silages from DIG and SBP to improve rumen fermentation, microbiota and nutrient degradability. Using in vitro and in vivo experiments, future studies may investigate a potential influence of the applied silage additives in diets with a higher silage proportion than in the present study.

## Ethics Statement

The authors confirm that the ethical policies of the journal, as noted on the journal's author guidelines page, have been adhered to. No ethical approval was required.

## Conflicts of Interest

The authors declare no conflicts of interest.

## Supporting information

Supporting information.

## Data Availability

All sequences have been submitted to the National Centre for Biotechnology Information sequence read archive and can be obtained under accession number PRJNA1150481. The R codes as well as the data that support the findings of this study are available from the corresponding author upon reasonable request.
