# Supplementary material for: An In Vitro Nutritional Evaluation of Mixed Silages of Drought‐Impaired Grass and Sugar Beet Pulp With or Without Silage Inoculants
Source: J Anim Physiol Anim Nutr (Berl). 2025 Jan 6;109(3):766–76. doi: 10.1111/jpn.14092 (PMC12091085; doi:10.1111/jpn.14092)
Supplement: Supplementary file 1 — Supporting information. [file JPN-109-766-s001.pdf]

An *in vitro* nutritional evaluation of mixed silages of drought-impaired grass and sugar beet pulp with or without silage inoculants

Theresa Gruber[1], Claudia Lang[1], Katerina Fliegerová[2], Georg Terler[3], Qendrim Zebeli[1], Thomas Hartinger[1]*

[1]Centre for Animal Nutrition and Welfare, University of Veterinary Medicine Vienna, Austria

[2]Institute of Animal Physiology and Genetics, Czech Academy of Sciences, Czech Republic

[3]Institute of Livestock Research, Agricultural Research and Education Centre Raumberg-Gumpenstein, Irdning-Donnersbachtal, Austria

*Correspondence: Thomas Hartinger thomas.hartinger@vetmeduni.ac.at

Supplementary

Supplementary Table 1. Chemical composition and silage fermentation characteristics of mixed silages prepared from drought-impaired grass and sugar beet pulp pellets with different silage additives (Gruber et al., 2024).

	Mixed silage[a]			
	CON	AF	RF	LAB
Chemical composition (g/kg DM[b] unless stated)				
DM concentration (g/kg)	356	355	362	350
Ash	82.2	84.8	82.2	82.5
Crude protein	109	109	104	103
Ether extract	19.8	20.3	17.2	18.7
aNDFom[c]	542	510	528	507
ADFom[d]	335	366	332	343
ADL[e]	64.6	125	60.0	61.6
Hemicelluloses[f]	207	144	195	164
Cellulose[g]	271	241	272	282
Water-soluble carbohydrates	80.5	34.8	80.5	49.7
Non-fiber carbohydrates	247	275	269	288
Silage fermentation characteristics (g/kg DM, unless for pH or otherwise noted)				
DM loss (%)	3.38	3.35	3.53	3.30
pH	3.73	3.86	3.79	3.72
Lactic acid	108	134	121	123
Acetic acid	4.32	10.5	12.1	12.8
Propionic acid	0.00	0.01	0.20	0.11
Butyric acid	n.d.[h]	n.d.	n.d.	n.d.
Ethanol	1.93	1.73	7.40	3.95
Ammonia-N (g/kg total N)	25.9	44.5	27.9	24.1

[a]Mixed silages prepared with drought-impaired grass and sugar beet pulp pellets (63:37 on DM basis) with or without different silage additives, i.e., without additive (CON), with fresh anaerobic fungi culture supernatant (AF), with fresh mixed ruminal fluid (RF), with lactic acid bacteria (LAB); [b]Dry matter; [c]Neutral detergent fibre assayed with a heat stable α-amylase and expressed exclusive of residual ash; [d]Acid detergent fibre expressed exclusive of residual ash; [e]Acid detergent lignin; [f]Calculated as aNDFom (g/kg DM) - ADFom (g/kg DM); [g]Calculated as ADFom (g/kg DM) - ADL (g/kg DM); [h]Not detected.

Supplementary Table 2. Chemical composition of the concentrate mixture comprised 66% of ground grain mixture and 34% of commercially available protein concentrate pellets.

	Ground grain mixture	Protein concentrate pellets
Chemical composition (g/kg DM[a] unless stated)		
DM concentration (g/kg)	897	899
Crude ash	86.5	93.2
Crude protein	113	444
Ether extract	23.7	30.7
aNDFom[b]	116	299
ADFom[c]	42.7	191
ADL[d]	15.3	77.6
Non-fiber carbohydrates	661	133

[a]Dry matter; [b]Neutral detergent fibre assayed with a heat stable α-amylase and expressed exclusive of residual ash; [c]Acid detergent fibre expressed exclusive of residual ash; [d]Acid detergent lignin.

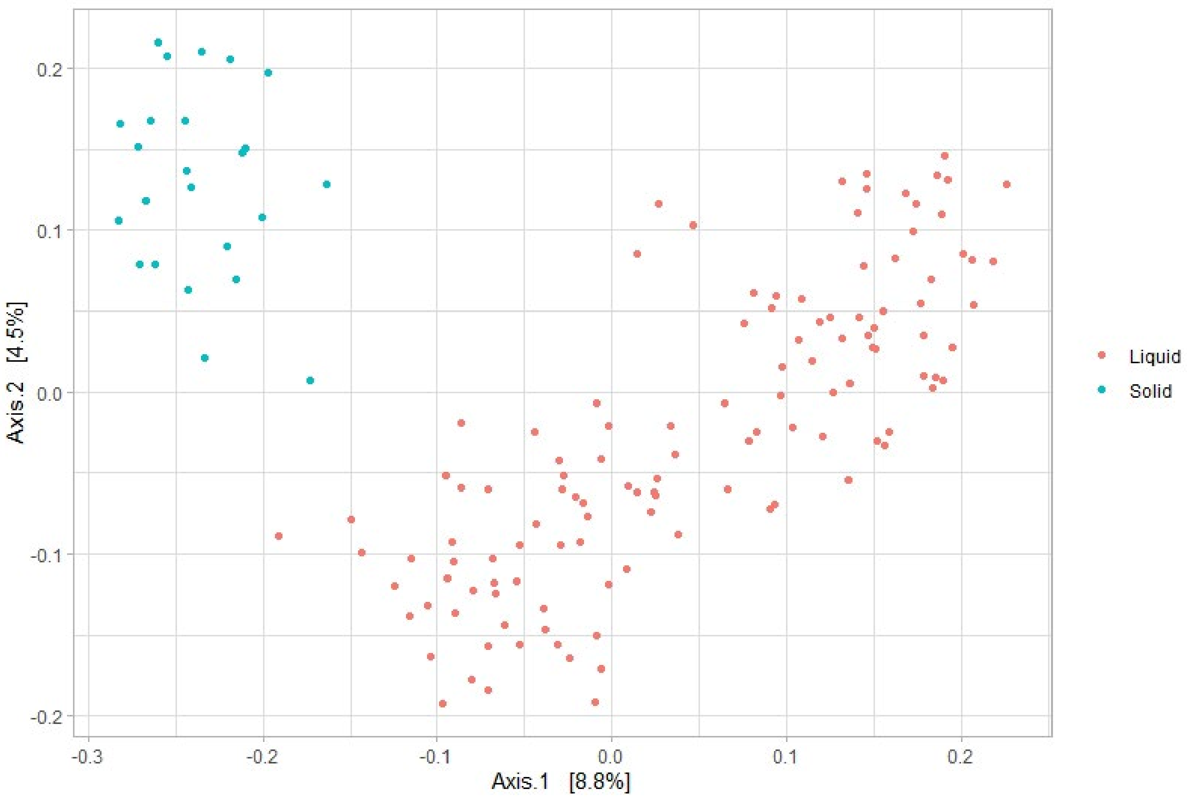

Supplementary Figure 1. Changes in microbial community composition associated with the liquid and solid fraction visualized as a principal co-ordinate analysis using weighted UniFrac distance metrics. The percentage of variation is indicated on the respective axes.

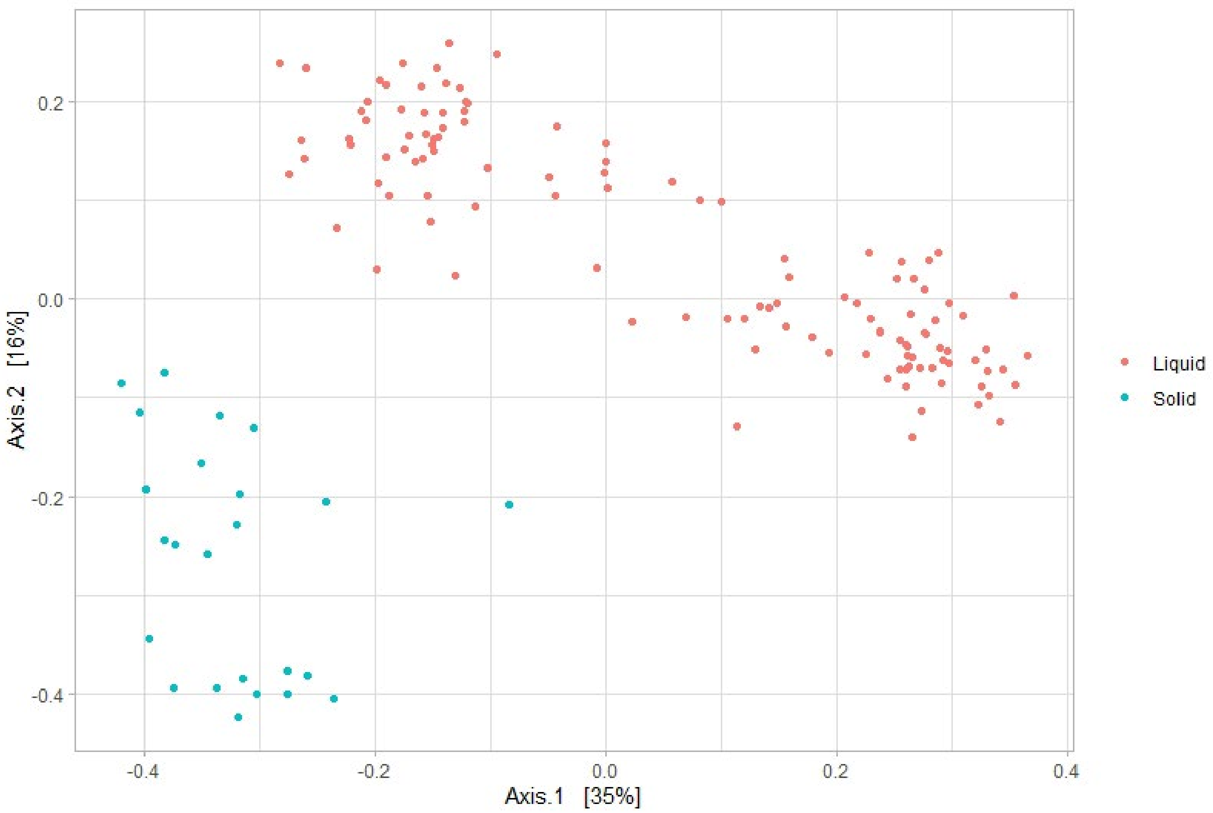

Supplementary Figure 2. Changes in microbial community composition associated with the liquid and solid fraction visualized as a principal co-ordinate analysis using Bray-Curtis distance metrics. The percentage of variation is indicated on the respective axes.

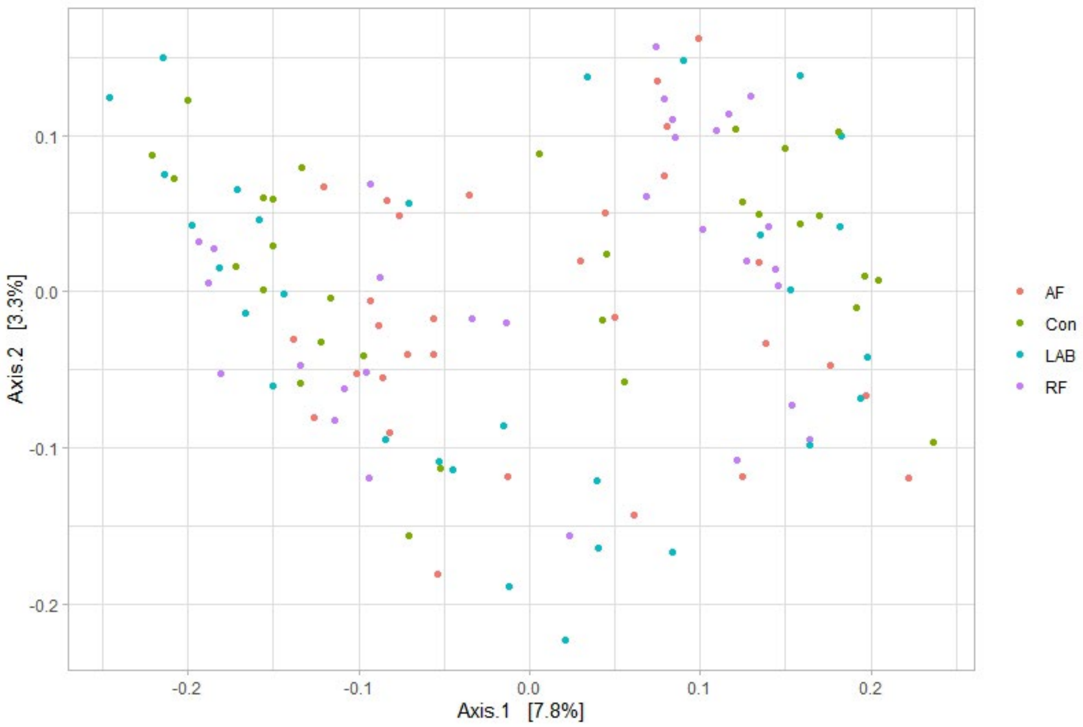

Supplementary Figure 3. Changes in microbial community composition in the liquid fraction associated with different treatment diets visualized as a principal co-ordinate analysis using unweighted UniFrac distance metrics. The percentage of variation is indicated on the respective axes. Treatment diets differed in the included silage of drought-impaired grass and sugar beet pulp pellets produced (i) without additive [CON], (ii) with fresh anaerobic fungi culture supernatant [AF], (iii) with fresh mixed ruminal fluid [RF], or (iv) with lactic acid bacteria [LAB].

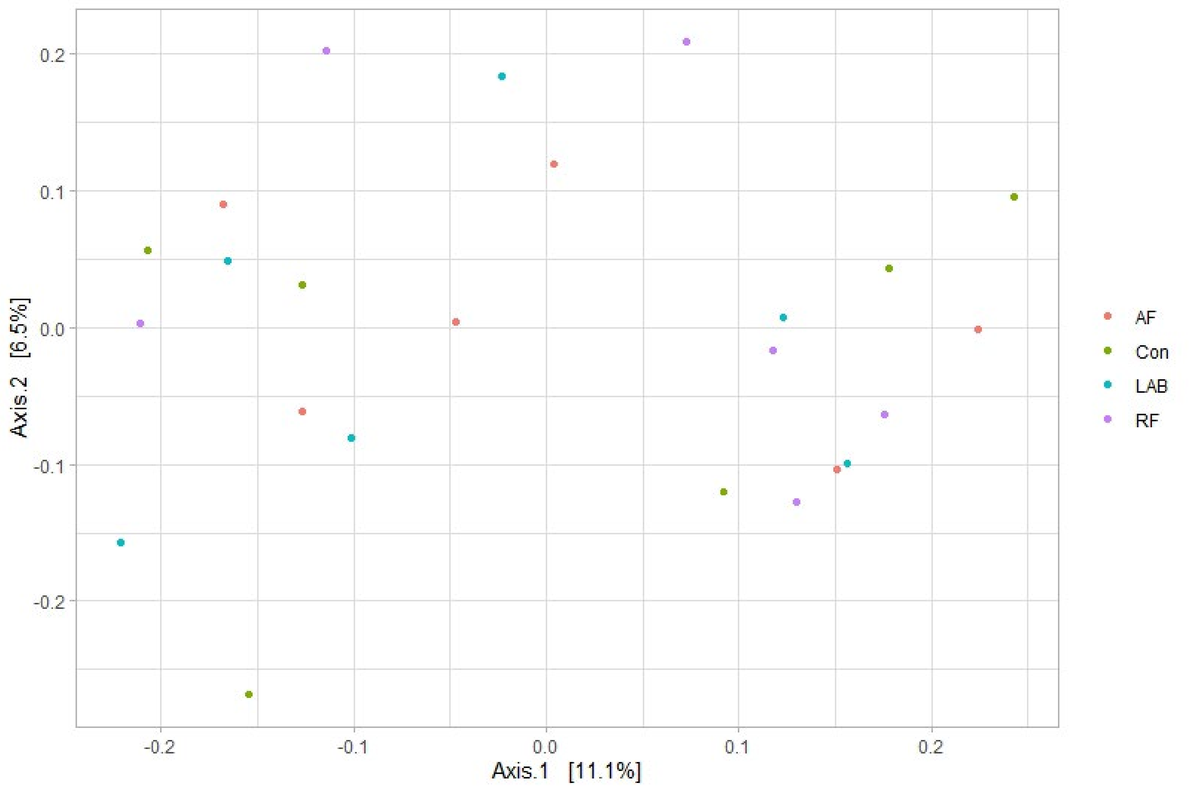

.

Supplementary Figure 4. Changes in microbial community composition in the solid fraction associated with different treatment diets visualized as a principal co-ordinate analysis using unweighted UniFrac distance metrics. The percentage of variation is indicated on the respective axes. Treatment diets differed in the included silage of drought-impaired grass and sugar beet pulp pellets produced (i) without additive [CON], (ii) with fresh anaerobic fungi culture supernatant [AF], (iii) with fresh mixed ruminal fluid [RF], or (iv) with lactic acid bacteria [LAB].

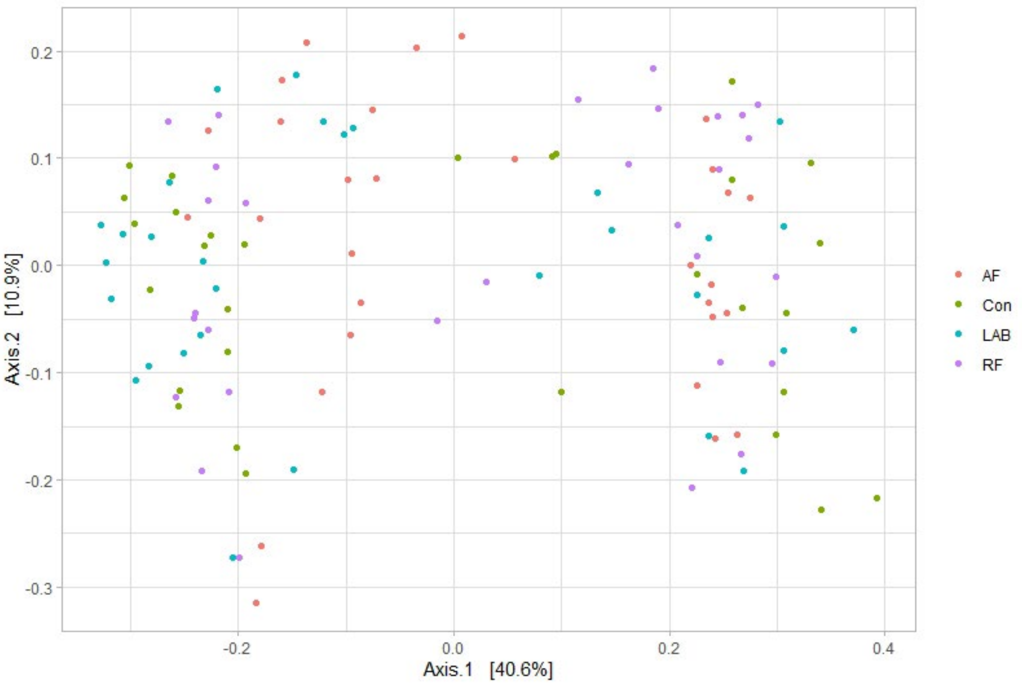

Supplementary Figure 5. Changes in microbial community composition in the liquid fraction associated with different treatment diets visualized as a principal co-ordinate analysis using Bray-Curtis distance metrics. The percentage of variation is indicated on the respective axes. Treatment diets differed in the included silage of drought-impaired grass and sugar beet pulp pellets produced (i) without additive [CON], (ii) with fresh anaerobic fungi culture supernatant [AF], (iii) with fresh mixed ruminal fluid [RF], or (iv) with lactic acid bacteria [LAB].

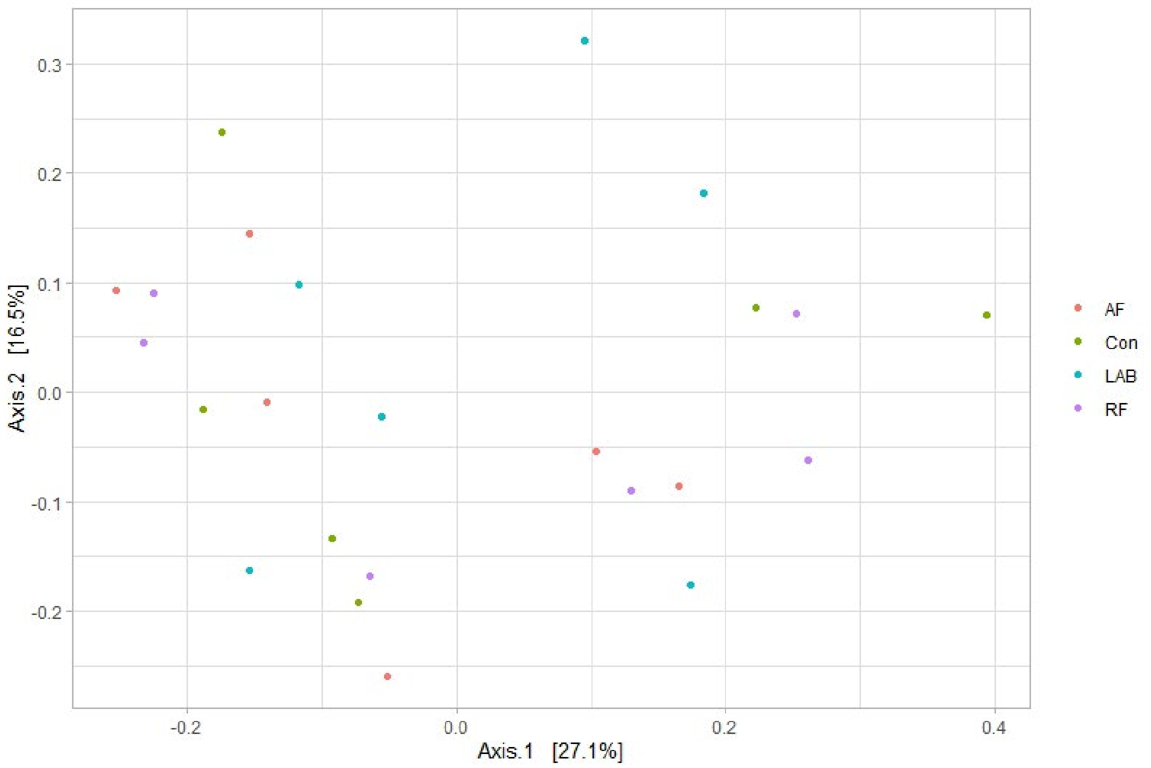

Supplementary Figure 6. Changes in microbial community composition in the solid fraction associated with different treatment diets visualized as a principal co-ordinate analysis using Bray-Curtis distance metrics. The percentage of variation is indicated on the respective axes. Treatment diets differed in the included silage of drought-impaired grass and sugar beet pulp pellets produced (i) without additive [CON], (ii) with fresh anaerobic fungi culture supernatant [AF], (iii) with fresh mixed ruminal fluid [RF], or (iv) with lactic acid bacteria [LAB].

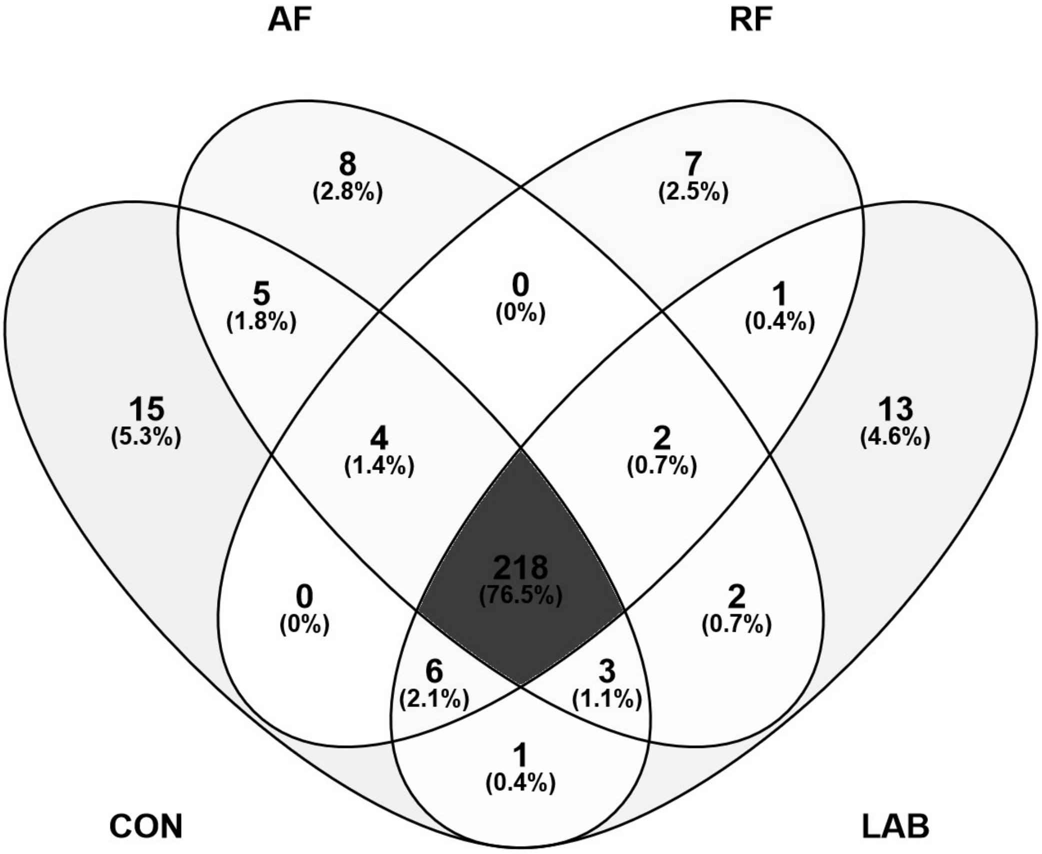

Supplementary Figure 7. Venn diagram illustrating the effect of treatment diets on the microbial composition at genus level in the liquid fraction. Treatment diets differed in the included silage of drought-impaired grass and sugar beet pulp pellets produced (i) without additive [CON], (ii) with fresh anaerobic fungi culture supernatant [AF], (iii) with fresh mixed ruminal fluid [RF], or (iv) with lactic acid bacteria [LAB].

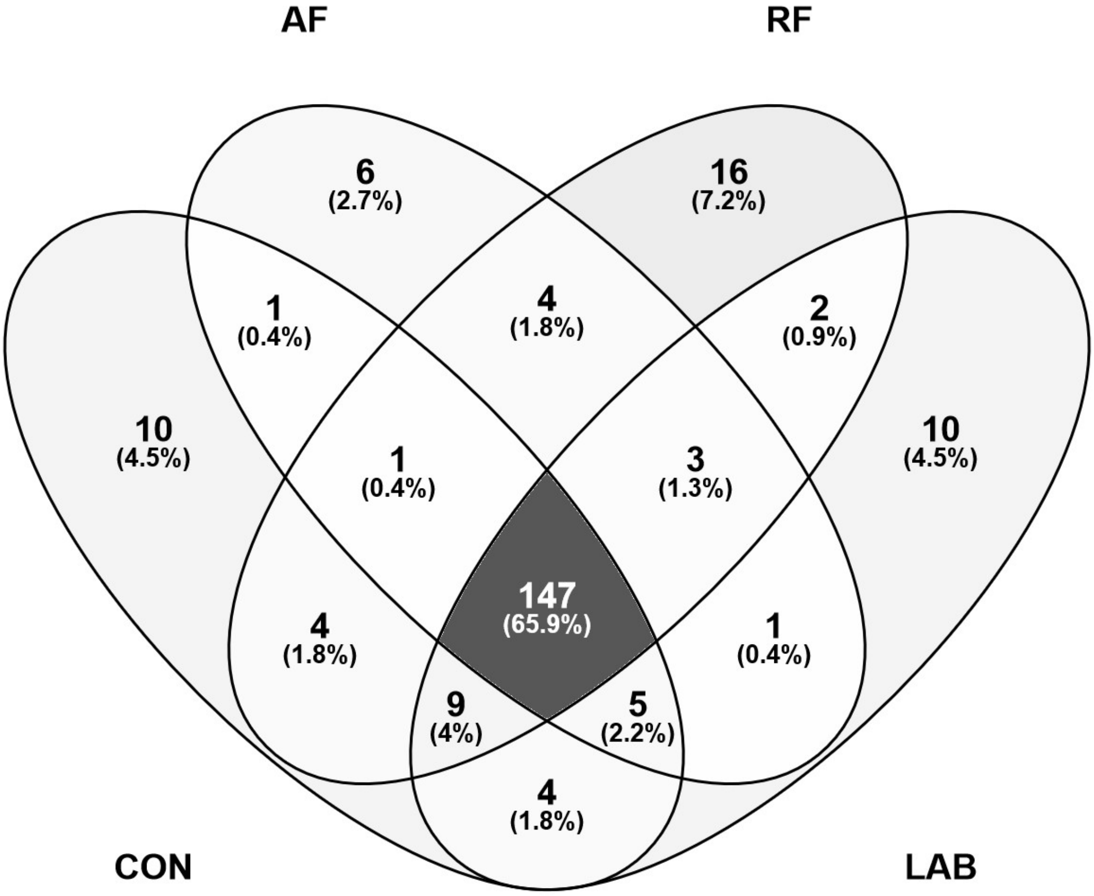

Supplementary Figure 8. Venn diagram illustrating the effect of treatment diets on the microbial composition at genus level in the solid fraction. Treatment diets differed in the included silage of drought-impaired grass and sugar beet pulp pellets produced (i) without additive [CON], (ii) with fresh anaerobic fungi culture supernatant [AF], (iii) with fresh mixed ruminal fluid [RF], or (iv) with lactic acid bacteria [LAB].
